# Altered alpha-band regulation during naturalistic theory of mind processing in autistic children

**DOI:** 10.1016/j.dcn.2026.101810

**Published:** 2026-09-01

**Authors:** Binbin Sun, Xuefei Pan, Tianqi Feng, Zhen Wei, Hong Wang, Shuo Zhao

**Affiliations:** a Shenzhen Maternity and Child Healthcare Hospital, Women and Children's Medical Center Southern Medical University, Shenzhen, Guangdong Province, China; b School of Psychology, Shenzhen University, Shenzhen, Guangdong, China; c Shenzhen Key Lab Affect and Social Neuroscience, Shenzhen University, Shenzhen, Guangdong, China; d Key Laboratory of Brain Cognition and Emotional Health of Guangdong Higher Education Institutes, Shenzhen University, Guangdong, China; e Intelligent Computing Center, Shenzhen University, Guangdong, China

**Keywords:** Theory of mind, Autistic children, Right temporoparietal junction, Electroencephalography, Alpha-band modulation

## Abstract

**Background:**

Theory of Mind (ToM) deficits are a core feature of autism, yet the neural dynamics associated with social-cognitive processing in naturalistic contexts remain unclear. Alpha-band oscillations have been implicated in the regulation of internal attention and may be involved in supporting mentalizing during real-world social interactions.

**Methods:**

Electroencephalography (EEG) was recorded from 41 autistic children and 34 typically developing (TD) children aged 3–10 years while they passively viewed a nonverbal animated film. Alpha power (6–9 Hz) in the right temporoparietal junction (rTPJ) and prefrontal cortex (PFC) was examined during Theory of Mind–related scenes and baseline scenes.

**Results:**

TD children showed increased alpha-band activity during ToM-related scenes relative to baseline, whereas autistic children showed no clear context-dependent alpha-band modulation. Exploratory ROI-level analyses further suggested associations between rTPJ alpha modulation and individual differences in ToMI-2 and Childhood Autism Rating Scale (CARS) scores, although these associations did not survive FDR correction.

**Conclusions:**

These findings suggest that typical ToM-related processing involves context-sensitive alpha-band modulation supporting social cognition. In autism, reduced modulation may reflect altered neural regulation during social-cognitive processing rather than diminished regional engagement. Naturalistic EEG paradigms may help identify neural indices of individual differences in social-cognitive functioning, pending further validation.

## Highlights

• A naturalistic movie EEG paradigm assessed alpha modulation during Theory of Mind processing in young children.
• Autistic children lacked context-dependent alpha modulation during ToM scenes observed in typically developing children.
• Exploratory rTPJ alpha associations with autism symptom severity require validation in independent samples.

## 1 Introduction

Autism is a neurodevelopmental disorder affecting more than 2% of 8-year-old children, with a comparable global prevalence (Ayoub, 2025). Social communication impairment is a core diagnostic feature of autism in the DSM-5 (Carter Leno et al., 2019), and theory of mind (ToM) difficulties are common in autism (Mangnus et al., 2024). For example, only 5% of autistic adolescents showed intact ToM performance on a false-belief task (Fadda et al., 2016), which may directly affect social interaction and communication (Yuk et al., 2020). Although behavioral ToM deficits in autism are well established, their neural mechanisms remain unclear, particularly whether atypical ToM processing reflects reduced social-brain engagement or disrupted dynamic regulation of internally oriented social cognition.

ToM is the ability to attribute mental states—such as beliefs, desires, and intentions—to oneself and others (Matthews and Goldberg, 2018). As a foundational aspect of children’s social development, ToM plays a vital role in social interaction, empathy, and interpersonal coordination (Maliske et al., 2023, Gan et al., 2024, Peristeri et al., 2024). In typically developing (TD) children, ToM development has been associated with the maturation and engagement of social brain networks, including the right temporoparietal junction (rTPJ) and prefrontal cortex (PFC) (Bowman et al., 2019, Symeonidou et al., 2020, Ghuman et al., 2017, Quiñones-Camacho et al., 2021). However, whether naturalistic ToM-related scenes elicit context-sensitive alpha-band enhancement in these regions remains an open empirical question. Notably, in developmental neuroscience, the alpha band for children is often defined within a lower frequency range, typically 6–9 Hz (Bick et al., 2019), rather than the conventional adult range of 8–13 Hz. This adjustment is crucial because the brain's peak alpha frequency is slower in childhood and increases with maturation (Klimesch, 1999, Finn et al., 2023). The functional role of alpha oscillations varies by brain region and task demands. In occipital and parietal areas, cognitive load often leads to alpha suppression, reflecting heightened sensory processing (Jensen and Mazaheri, 2010, Klimesch, 2012, Su et al., 2020).

Conversely, alpha enhancement in anterior regions like the PFC and rTPJ is believed to reflect a shift to internal attention and the inhibition of irrelevant sensory input (Magosso et al., 2019, Sokoliuk et al., 2019). Beyond reflecting task engagement, alpha-band oscillations are increasingly understood as a regulatory mechanism that gates sensory input and supports internally oriented cognitive processing. Such regulation may be particularly critical for Theory of Mind, which requires the construction and updating of internal models of others’ mental states in dynamic social environments. Consequently, increased alpha-band modulation in TD children during ToM tasks may signal the suppression of external distractions to support complex mentalizing (Zhou et al., 2023). However, are ToM deficits in autistic children accompanied by specific, measurable atypicalities in alpha-band activity? More precisely, what are the differences in alpha oscillation patterns between autistic children and their TD peers? And could these neural differences serve as an effective neural marker to characterize, or even predict, the severity of their real-world social difficulties?

Previous investigations into the neural correlates of ToM in autism have predominantly relied on standardized laboratory tasks, such as the false belief task. However, this approach faces two significant limitations. Firstly, the rigid and often abstract instructions of these tasks can be challenging for autistic children to follow, making effective implementation difficult and potentially confounding the results. Secondly, such tasks often fail to capture the complexity and dynamic nature of real-world social interactions, thus limiting their ecological validity in assessing ToM deficits (Atherton et al., 2019, Mangnus et al., 2024). This disconnect is highlighted by the observation that autistic children may succeed in structured ToM tasks yet still experience persistent difficulties in everyday social situations (Peterson et al., 2009), suggesting that traditional paradigms may underestimate their real-world challenges.

To address these issues, there is a compelling need to employ naturalistic stimuli, such as the animated film "Partly Cloudy", to probe the neural signatures of ToM in children (Peng et al., 2026, Richardson et al., 2018). A primary challenge in this area, however, has been the reliance on neuroimaging methods like fMRI(Nastase et al., 2021). The fMRI environment is particularly problematic for autism research; its requirement for children to remain completely still within a noisy, restrictive machine makes successful data collection especially difficult and often impossible for this group. As a more suitable tool for this research, EEG is far more tolerant of participant movement. For this reason, leveraging EEG with ecologically valid, naturalistic stimuli paradigms is crucial for meaningfully advancing our understanding of the real-world neural mechanisms of ToM difficulties in autism.

This study employs an ecologically valid, naturalistic movie-viewing paradigm, using the animated short film "Partly Cloudy", in conjunction with electroencephalography (EEG). We aimed to examine whether 6–9 Hz alpha-band activity during naturalistic viewing reflects the dynamic neural regulation of internally oriented social cognition, and whether disruptions in this regulation characterize autistic children. Specifically, we want to determine if alpha-band activity during naturalistic viewing can serve as a reliable neural marker for real-world ToM abilities, capable of distinguishing between TD children and autistic children**.** We also seek to understand if these neural differences could effectively characterize, or even predict, the severity of real-world social difficulties in autistic children. We hypothesize the following: In TD children, we expect that ToM-related conditions will elicit enhanced alpha-band modulation in key brain regions like the PFC and rTPJ. If observed, such enhancement would be consistent with a shift toward internally oriented attention and the inhibition of irrelevant stimuli (Magosso et al., 2019, Sokoliuk et al., 2019), and we examined whether this modulation was associated with Theory of Mind Inventory-2 (ToMI-2) scores, a measure of everyday ToM abilities.

In autistic children, we hypothesized that they would show atypical context-dependent alpha-band modulation during ToM-related scenes, rather than the ToM-related increase in alpha-band modulation expected in TD children. Furthermore, we predict that the extent of this atypical alpha activity in the ^autistic group^ will correlate with the severity of their autism-related symptoms. By testing these hypotheses, this study seeks to elucidate the neural mechanisms of ToM in autism within a naturalistic context and to identify a potential alpha-based neural marker for real-world social-behavioral deficits. A successful outcome would provide a theoretical foundation for the automated identification of ToM abilities, inform the development of objective potential feature in multivariate characterization, and highlight a potential target for clinical interventions. Examining these neural regulatory mechanisms during early childhood may provide insights into how social-cognitive functions emerge and diverge across development.

## 2 Methods

### 2.1 Participants

According to the calculation of G*Power3.1 (Faul et al., 2007), intra-group repeated measurement ANOVA was used in this experiment, and the total sample size was predicted to be at least 36 when the statistical test power (1-β) was 0.95 at the significance level of α = 0.05 and the effect was moderate (*f* = 0.25).

**Autistic group:** Forty-one autistic children were recruited through collaboration with the Department of Child Psychology and Rehabilitation at Shenzhen Maternity and Child Healthcare Hospital. Inclusion criteria were: (1) Age 3–10 years; (2) Diagnosis of autism confirmed according to DSM-5 criteria; (3) No history of epilepsy or head trauma; (4) No astigmatism, color weakness, or color blindness; (5) IQ scores > 70. Clinical characterization of the autistic group. Autism diagnoses were established by experienced clinicians according to DSM-5 criteria. Autism-related symptom severity was characterized using the Childhood Autism Rating Scale (CARS), and ADOS scores were used to provide additional standardized clinical characterization of the autistic group. CARS scores were used as the primary continuous measure of autism-related symptom severity in the brain–behavior analyses, whereas ADOS scores were reported descriptively and were not included in the primary correlation analyses. Information on medication status, language ability, intervention history, and major neurological or psychiatric comorbidities was obtained from caregiver report and clinical records when available. Children with a history of epilepsy, head trauma, or severe uncorrected sensory impairments were excluded. All included children had an estimated full-scale IQ above 70.

Control Group: Thirty-four children with TD were recruited under the same institutional collaboration. Inclusion criteria comprised: (1) Age 3–10 years; (2) No diagnosis of neurodevelopmental/psychiatric disorders; (3) No epilepsy or head trauma history; (4) No color weakness/blindness, astigmatism < 100 °; (5) IQ scores > 70.

All experimental procedures and participant rights were fully explained to children and their guardians. Guardians provided written informed consent and received comprehensive assessment reports along with transportation reimbursements after completing all study protocols.

Group-specific demographic and available clinical characteristics are summarized in Table 1.

**Table 1:** Demographic information.

Characteristics	TD (n = 34)	Autistic (n = 41)	t/χ²	p value
^Age, *M(SD), y*^	^7.59(1.47)^	^6.98(1.43)^	^1.81^	^0.075^
^[range]^	^[4.16−10.50]^	^[3.83−10.91]^	^-^	^-^
^FSIQ, *M(SD)*^	^101.38(10.72)^	^100.41(15.75)^	^0.30^	^0.762^
^[range]^	^[73−120]^	^[78−129]^	^-^	^-^
^Gender, *n(%)*^	^-^	^-^	^5.07^	^0.030^
^Girls^	^9(26.47%)^	^3(7.32%)^	^-^	^-^
^Boys^	^25(73.53%)^	^38(92.68%)^	^-^	^-^
^TOMI−2, *M(SD)*^	^17.82(0.33)^	^12.18(0.47)^	^8.72^	^0.000^
^ADOS, *M(SD)*^	^-^	^14.24(3.25)^	^-^	^-^
^CARS, *M(SD)*^	^-^	^34.11(3.02)^	^-^	^-^

### 2.2 Stimuli and experimental setting

**Fig. 1:**
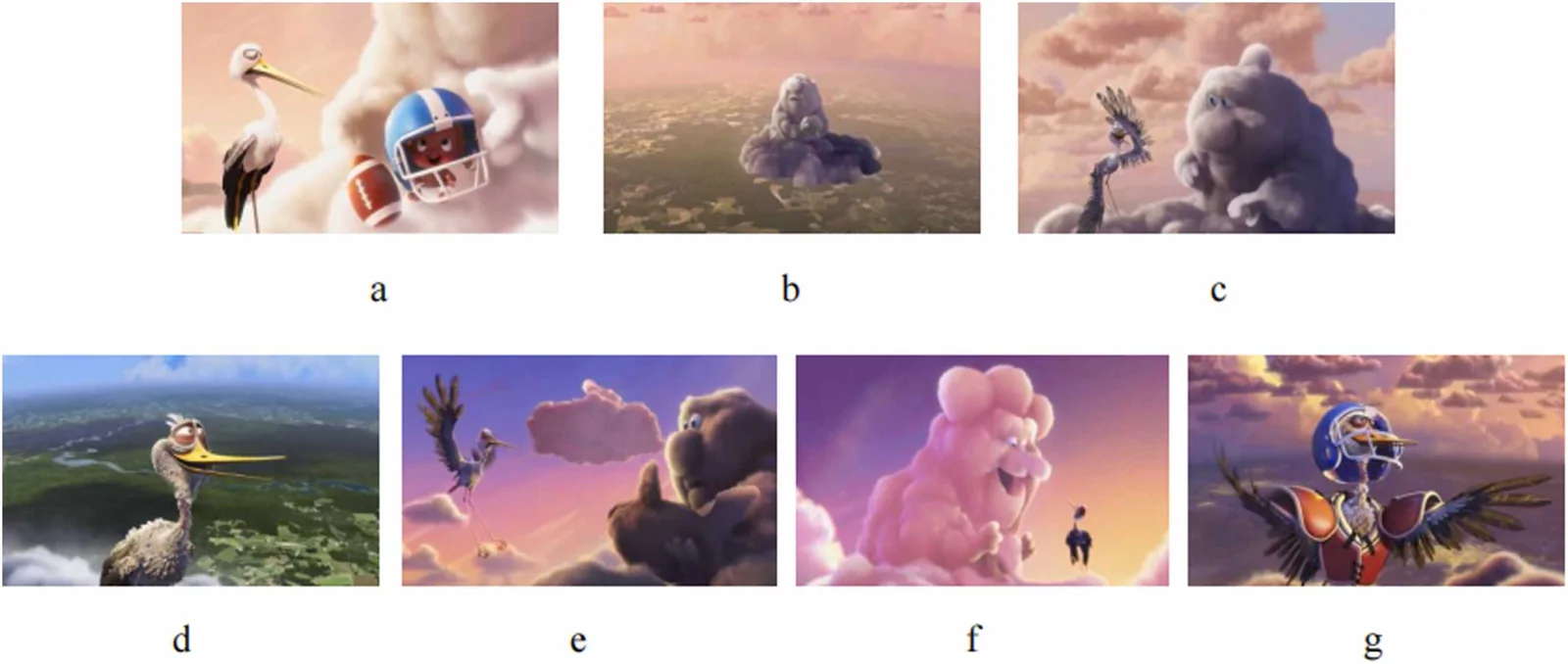
Seven instances of Theory of Mind (ToM) events defined in the animated short film Partly Cloudy. (a)A crying baby becomes joyful upon receiving a helmet; (b)the scene shifts from white clouds to the isolated-looking storm cloud Gus; (c)the stork Peck and Gus (friends) exchange greetings; (d)Peck gazes longingly at a neighboring cloud crafting puppies, prompting Gus to appear apprehensive, after which Peck notices his own envy and grows embarrassed; (e)Peck startles at the shark baby created by Gus and observes another cloud producing adorable chicks; (f)Peck flies away from Gus, landing on a nearby cloud that seems to mock Gus; (g)Peck dons protective gear to demonstrate loyalty to Gus, requesting the cloud to craft a protective suit for himself. Peek returns to Gus in protective gear, showing through his actions that he did not leave.

Children watched the non-verbal animated film "Partly Cloudy" in an electromagnetically shielded and soundproofed room. The film has a duration of approximately 5 min, and its brief plot summary is publicly available online (https://www.pixar.com/partly-cloudy#partly-cloudy-1). This stimulus was chosen for its feasibility with children, as it is short, engaging, and requires no explicit learning tasks. The film has been validated to engage ToM-related brain regions in both adults (Jacoby et al., 2016) and children (Richardson et al., 2018) (see Fig. 1). A 10-second rest period preceded stimulus presentation. Children were instructed to watch the film passively while remaining still.

The movie stimulus was segmented into distinct event categories based on the coding scheme established by Richardson et al. (2018). Three primary event types were identified for this study: ToM Events: Scenes requiring the inference of complex mental states (e.g., a character misinterpreting a social cue and believing they are abandoned). Pain Events: Scenes depicting clear instances of physical harm or distress (e.g., a character getting injured). Other Events: All remaining segments that were not categorized as either ToM or Pain (e.g., background scenery like birds flying, simple character movements).

From these event types, two main conditions were constructed for the primary analysis investigating ToM. The ToM Condition consisted of all defined ToM Events. The Baseline Condition was created as a composite of all 'Other Events,' thereby explicitly excluding segments with higher-order ToM or pain-related content. The rationale for this baseline construction was to create a robust control condition. This baseline captures a wide range of perceptual and cognitive processes present throughout the film (such as general visual processing, attention, and observation of simple actions) that are not specific to complex mentalizing. By contrasting the ToM condition against this comprehensive baseline, our analysis aimed to isolate the neural activity specifically associated with the act of mental state attribution, effectively subtracting out the influence of these lower-level, non-specific processes. The specific time division of each condition is presented in the supplementary Table S1.

### 2.3 Measurement

*Childhood Autism Rating Scale (CARS).* The CARS is a clinician-administered, observational assessment of autism-related symptoms in children that is recognized worldwide for its standardized criteria and diagnostic utility (Lord and Corsello, 2005). Validated for children 18 months and older, it requires professional expertise to administer and provides objective diagnostic insight through behavioral analysis. The CARS demonstrates strong internal consistency, with Cronbach’s alpha values ranging from 0.82 to 0.95 (Moon et al., 2019). The CARS assessment, which consists of 15 items scored on a 1–4 scale (∼30 min), evaluates social interaction, communication, restricted/stereotyped behaviors, sensory responsiveness, and intellectual functioning. Total scores (max 60) use a tiered cutoff: ≤ 30 rules out autism; 30–37 indicates mild to moderate autism; and ≥ 37 (with ≥5 items >3) indicates severe autism (Geier et al., 2013). This quantifiable framework provides a standardized diagnostic lens for the severity of autism-related symptoms.

***Autism Diagnostic Observation Schedule (ADOS).*** The ADOS is a semi-structured, standardized clinical observation tool for assessing individuals suspected of having autism. It evaluates core autism-related behaviors in social communication, interaction, and restricted or repetitive behaviors through standardized social interaction tasks, with modules selected according to language ability and developmental level. Administered by certified professionals, the ADOS is widely regarded as part of the diagnostic “gold standard” for autism and has demonstrated strong sensitivity, specificity, inter-rater reliability, and internal consistency (Falkmer et al., 2013, Kamp-Becker et al., 2018, Barbaresi et al., 2022, Moffitt et al., 2022). Its calibrated severity scores also provide quantitative indices for symptom characterization and tracking clinical change (Bieleninik et al., 2017).

*IQ measurement.* Intellectual functioning was assessed using the Chinese-adapted short forms of the Wechsler Preschool and Primary Scale of Intelligence-Fourth Edition and the Wechsler Intelligence Scale for Children-Fourth Edition (WPPSI-IV-SF and WISC-IV-SF; Wechsler, 2003, Wechsler, 2012; Fan et al., 2019). The WPPSI-IV is appropriate for children aged 2 years 6 months to 7 years 7 months, whereas the WISC-IV is designed for children aged 6 years to 16 years 11 months. Both instruments provide estimates of full-scale IQ (FSIQ) and domain-level cognitive abilities through standardized subtests. To reduce participant burden while retaining reliable FSIQ estimation, abbreviated Chinese versions were administered, consistent with prior work supporting short-form Wechsler assessments in clinical and pediatric populations (Axelrod, 2002, Piovesana et al., 2019). The WPPSI-IV-SF included six subtests: Information, Similarities, Block Design, Matrix Reasoning, Picture Concepts, and Bug Search. The WISC-IV-SF included four subtests: Digit Span, Similarities, Matrix Reasoning, and Coding. The Chinese WISC-IV-SF has been shown to provide reliable and valid cognitive estimates in both typically developing children and autistic children (Lin et al., 2017).

*Theory of Mind Inventory−2(ToMI-2).* The ToMI-2 is a psychological instrument designed for the assessment of an individual's capacity to comprehend others' mental states in complex social scenarios. The scale comprises 60 caregiver-reported items, which are designed to assess a range of abilities including emotional understanding, belief reasoning, and intent judgment. The items are presented as statements, such as "My child can understand whether others hurt him intentionally or accidentally", with scores ranging from 0 to 20. Scores on the test are positively correlated with the extent of the subject's ToM abilities.In children diagnosed with TD, the test demonstrates excellent test-retest reliability (r = 0.89) and strong construct validity (ρ = 0.66, with behaviour-based ToM measures). The internal consistency of the scale in this study was high (Cronbach's alpha = 0.98).

### 2.4 EEG recording, reduction, and analyses

EEG data was collected using a 64-channel HydroCel Geodesic Sensor Net (EGI, Eugene, OR, USA). The signal was recorded continuously and digitized at a 500 Hz sampling rate, using the vertex sensor (Cz) as the recording reference and applying an online 100 Hz low-pass filter. Scalp impedance for each sensor was maintained below 50 kΩ. Following data recording, EEG data processing was performed offline using MATLAB (version 2023a, MathWorks) with 50 Hz-notch and band pass filters (0.1–100 Hz). The EEGLAB toolbox (Delorme and Makeig, 2004) was used for offline preprocessing. After visual inspection and removal of segments containing gross movement artifacts, independent component analysis (ICA) was performed to identify stereotyped artifacts. Components were rejected if their scalp topographies, time courses, and power spectra were consistent with ocular artifacts, horizontal eye movements, muscle activity, or non-physiological noise. Component rejection was performed by trained researchers and checked for consistency before back-projecting the remaining components to the sensor space. EEG data were converted to the average reference. Power Spectral Densities (PSD) were derived by means of a discrete Fourier transform with a Hamming window (50% overlap) for each epoch within each condition type (Baseline, ToM) for each channel for each child participant, so that EEG findings were specific to both Baseline and ToM condition types. To ensure comparable data quantities across conditions, a fixed number of 59 overlapping 1-s epochs was extracted for each condition and participant whenever sufficient artifact-free data were available. The PFC and rTPJ ROIs were defined a priori based on prior evidence implicating the rTPJ and PFC in mentalizing and social-cognitive regulation (Bowman et al., 2019, Ghuman et al., 2017, Symeonidou et al., 2020, Quiñones-Camacho et al., 2021). Because scalp EEG provides limited spatial specificity, electrode clusters were selected as approximate scalp-level representations of these regions, following prior EEG studies using frontal and temporoparietal electrode groupings (Dallmer-Zerbe et al., 2020, Yang et al., 2021). The rTPJ ROI included T8, P8, and TP8 to approximate right posterior temporal–inferior parietal scalp activity, whereas the PFC ROI included F1, F2, AF3, and AF4 to capture bilateral anterior frontal activity. Absolute mean power within the alpha power (6–9 Hz) was calculated for subsequent analyses. For each ROI, alpha-band modulation (Δα) was calculated as the difference in average alpha-band modulation of the selected electrodes between the two condition types (α[ToM]-α[Baseline]).

### 2.5 Statistical analysis

Statistical analyses were conducted using SPSS 26.0 with a two-sided test, and statistical significance was set at *p* < 0.05. Firstly, the normality of the data was checked. If the data were normally distributed, a three-way analysis of covariance (ANCOVA) was performed on the average alpha-band modulation, with the within-subject factors of condition type (Baseline/ToM) and ROI (PFC/rTPJ), and the between-subjects factor of group (TD/autistic). Based on Social baseline theory −the baseline of human behavior is not a single state, but can contain multiple coexisting conditions, social conditions represent familiar or predictable social interactions that correspond to optimal human energy efficiency and emotional regulation, neutral condition usually represents the no-intervention control or minimal stimulus state and is used as a reference point to isolate variable effects (Gross and Medina-DeVilliers, 2020)−we combined the neutral and social conditions as Baseline conditions. This setup is consistent with the purpose of this study, and captures the natural state of human beings more comprehensively.

Gender was included as a covariate in the ANCOVA model. Brain–behavior associations were examined separately within each participant group. For these analyses, ROI-level alpha-band modulation was defined as Δα = α[ToM] − α[Baseline] for the PFC and rTPJ ROIs. Behavioral measures included ToMI-2 scores in both groups and CARS scores in the autistic group.

Before conducting the correlation analyses, normality was assessed for each behavioral measure and ROI-level alpha-band modulation variable. Pearson correlations were used when both variables were normally distributed, whereas Spearman rank correlations were used when either variable violated the normality assumption. Correlation coefficients are reported as effect sizes, together with exact uncorrected *p* values and 95% confidence intervals. For all inferential tests, exact p values are reported where possible. Partial eta squared (*η*_*p*_*²*) was reported as the effect size for ANCOVA effects. To account for multiple testing in the brain–behavior analyses while preserving the a priori structure of the hypotheses, Benjamini–Hochberg false discovery rate (FDR) correction was applied separately within each participant group and behavioral domain across the two a priori ROIs. Specifically, ToMI-2 correlations in the TD group were corrected across rTPJ and PFC. In the autistic group, CARS correlations and ToMI-2 correlations were corrected separately across rTPJ and PFC. Both uncorrected p values and FDR-adjusted q values are reported.

## 3 Results

### 3.1 Analysis of covariance

**Fig. 2:**
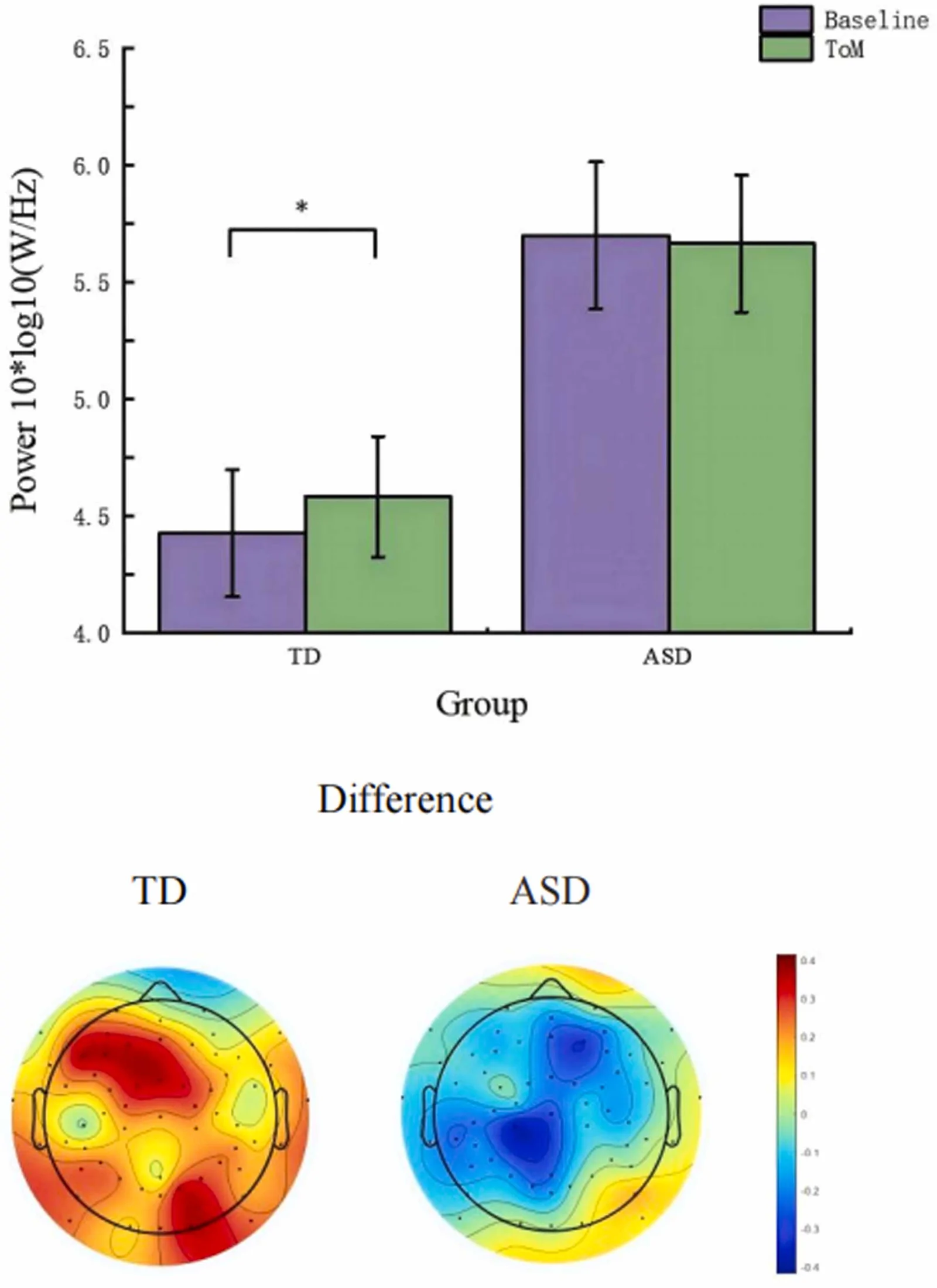
Alpha-band modulation in TD and autistic group. (a) Alpha power differences between conditions and groups. (b) Topographic maps illustrate the whole−brain averaged alpha-band modulation spectral density under the difference between baseline and ToM conditions in TD and autistic group. *p < 0.05.

According to the results of the normality test, all data conformed to a normal distribution, allowing further three-way analysis of covariance (ANCOVA) (Fig. 2). The ANCOVA results showed a significant main effect of group，*F*(1, 73) = 6.56, *p* = 0.013, *η*_p_² = 0.08, indicating that the averaged alpha-band modulation was higher in the autistic group than in the TD group. The interaction between condition and group was significant, *F*(1, 73) = 4.00, *p* = 0.037, *η*_p_² = 0.06. However, the Condition × ROI × Group interaction was not significant, *F*(1, 73) = 0.64, *p* = 0.427, *η*_p_² = 0.01. ROI × Group interaction was not significant, *F*(1, 73) = 1.82, *p* = 0.182, *η*_p_² = 0.02. Condition × ROI interaction was not significant, *F*(1, 73) = 1.05, *p* = 0.309, *η*_*p*_² = 0.01. The main effect of Condition was not significant, *F*(1, 73) = 1.87, *p* = 0.176, *η*_*p*_² = 0.03. The main effect of ROI was not significant, *F*(1, 73) = 3.71, *p* = 0.058, *η*_*p*_² = 0.05.

Post-hoc analyses were conducted to further clarify the significant interaction between condition and group. To control for multiple comparisons across the four pairwise tests, we applied Benjamini-Hochberg FDR correction (*q* < 0.05). In the TD group, alpha-band modulation was significantly higher during the ToM condition than during the Baseline condition (*p* = 0.021, q = 0.028), indicating enhanced alpha-band activity when processing ToM-related scenes. In contrast, in the ^autistic group^, no significant difference in alpha-band modulation was observed between the ToM and Baseline conditions (*p* = 0.573, *q* = 0.573). These results suggest that modulation of alpha-band activity by ToM-related content was present in TD children but absent in autistic children.

### 3.2 Correlational analyses

**Fig. 3:**
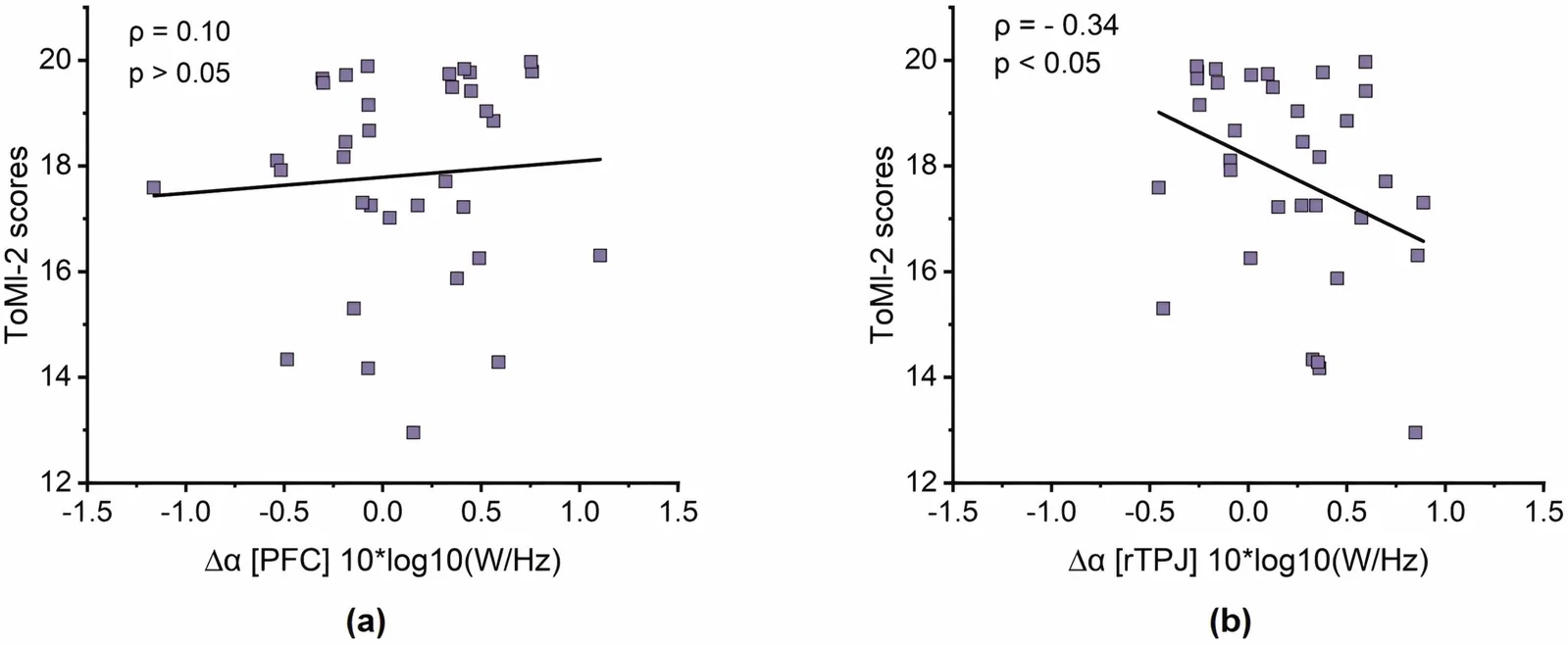
Correlation analysis in group TD(N = 34). (a) Association between ToMI-2 scores and PFC alpha modulation. (b) Association between ToMI-2 scores and rTPJ alpha modulation. The rTPJ association was negative in direction.

Before the correlation analyses, normality tests were performed separately for behavioral measures and ROI-level alpha-band modulation variables in the TD and ^autistic group^s. In the TD group, ToMI-2 scores were not normally distributed (*p* = 0.003), whereas all alpha-band modulation variables were normally distributed (*ps* > 0.406). Therefore, Spearman rank correlations were used for the associations between ToMI-2 scores and alpha-band modulation in the two a priori ROIs. In the ^autistic group^, rTPJ alpha-band modulation was not normally distributed (*p* = 0.028), whereas the remaining behavioral and alpha-band modulation variables were normally distributed (*ps* > 0.077). Accordingly, Spearman rank correlations were used for analyses involving rTPJ alpha-band modulation, whereas Pearson correlations were used for analyses involving PFC alpha-band modulation when both variables met the normality assumption. For TD children (Fig. 3), ToMI-2 scores were not normally distributed; therefore, Spearman rank correlations were used to examine the associations between ToMI-2 scores and alpha-band modulation in the two a priori ROIs. ToMI-2 scores showed a negative association with rTPJ alpha-band modulation, *ρ*= −0.34, 95% CI [−0.65, −0.03], uncorrected *p* = 0.049, FDR-adjusted q = 0.098. In contrast, no significant association was observed between ToMI-2 scores and PFC alpha-band modulation, *ρ*= 0.10, 95% CI [−0.25, 0.46], uncorrected *p* = 0.563, FDR-adjusted q = 0.563. Considering the multiple-comparison correction, the rTPJ association should be interpreted cautiously as an exploratory brain–behavior association.

**Fig. 4:**
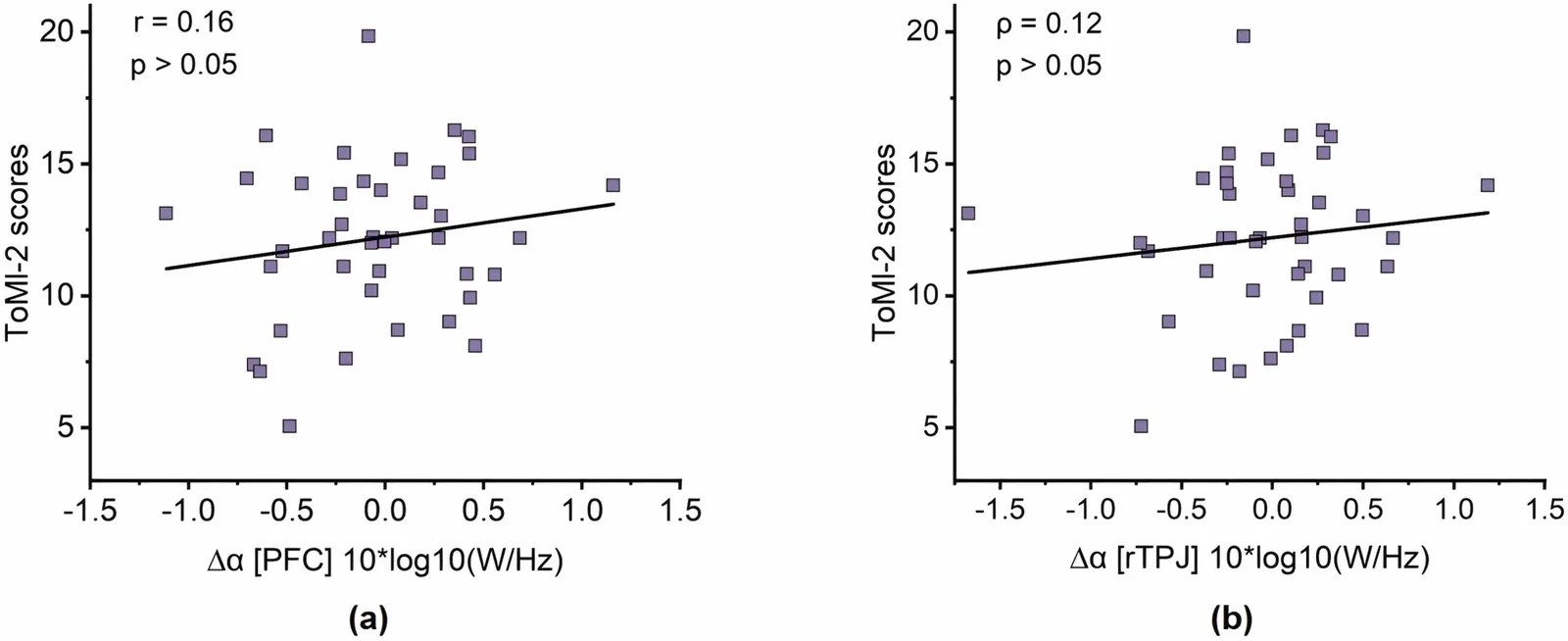
Correlation analysis in autistic group (N = 41). (a) correlation between ToMI−2 scores and alpha-band modulation in PFC; (b) correlation between ToMI−2 scores and alpha-band modulation in rTPJ.

**Fig. 5:**
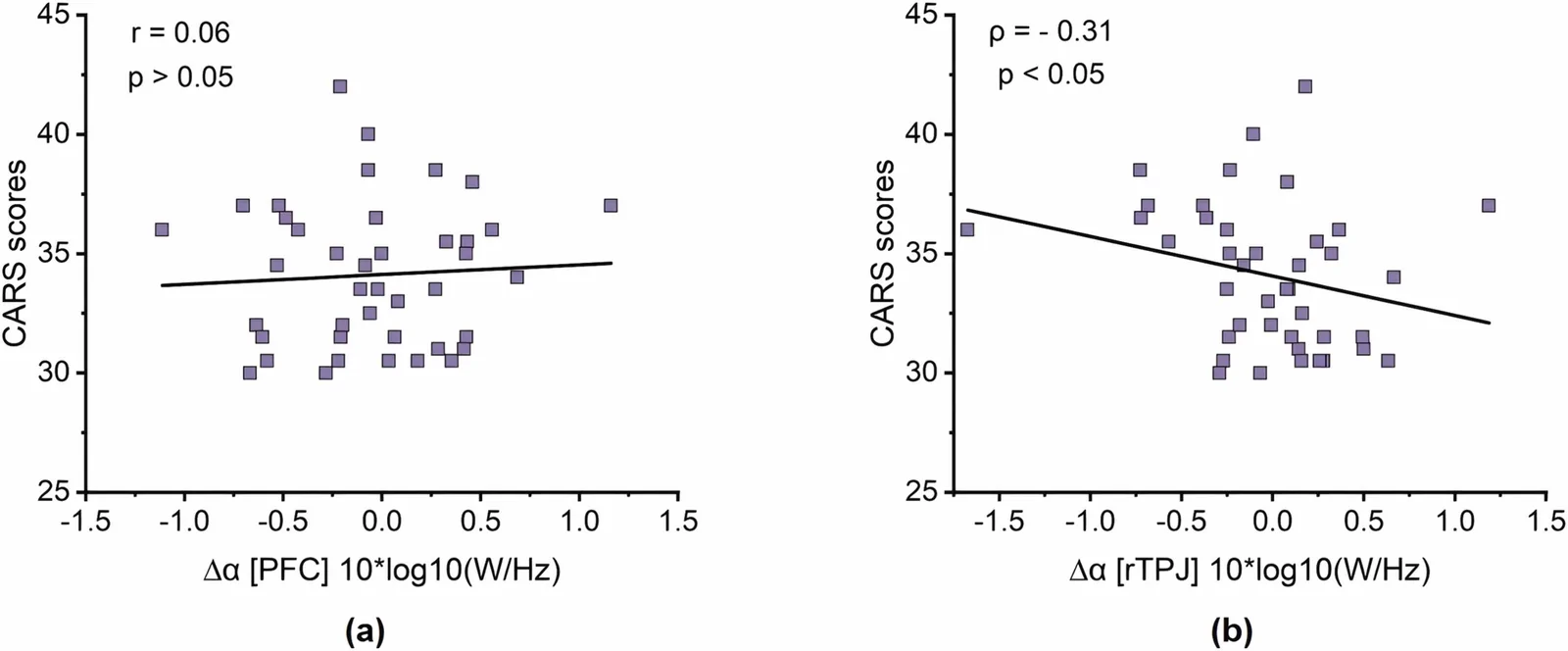
Correlation analysis in autistic group (N = 41). (a) correlation between CARS scores and alpha-band modulation in PFC; (b) correlation between CARS scores and alpha-band modulation in rTPJ. The rTPJ association was negative in direction.

In the autistic group, rTPJ alpha-band modulation was not normally distributed; therefore, Spearman rank correlations were used for analyses involving rTPJ alpha-band modulation. For ToMI-2 scores, no significant association was observed with PFC alpha-band modulation, *r* = 0.16, 95% CI [−0.12, 0.43], uncorrected *p* = 0.311, FDR-adjusted *q* = 0.622 (Fig. 4a), or with rTPJ alpha-band modulation, *ρ* = 0.12, 95% CI [−0.18, 0.40], uncorrected *p* = 0.440, FDR-adjusted *q* = 0.622 (Fig. 4b). For CARS total scores, no significant association was observed with PFC alpha-band modulation, *r* = 0.06, 95% CI [−0.20, 0.35], uncorrected *p* = 0.703, FDR-adjusted *q* = 0.703 (Fig. 5a). In contrast, rTPJ alpha-band modulation showed a negative association with CARS total scores, *ρ* = −0.31, 95% CI [−0.60, 0.02], uncorrected *p* = 0.047, FDR-adjusted *q* = 0.094 (Fig. 5b). Considering the multiple-comparison correction, the rTPJ–CARS association should be interpreted cautiously as exploratory.

## 4 Discussion

The ecological validity of research paradigms is a critical concern in studying ToM, a core mechanism of social-cognitive development. Here, we addressed this issue by testing ToM-related neural responses in a naturalistic movie-viewing context. In TD children, rTPJ alpha-band modulation showed an exploratory negative association with caregiver-reported ToM abilities, suggesting that alpha activity elicited by the paradigm may relate to individual differences in everyday social-cognitive functioning. However, this association should be interpreted as a correlational and non-specific neural index rather than evidence that stronger rTPJ alpha modulation directly reflects better ToM ability. Consistent with our hypothesis, autistic children showed reduced context-dependent alpha-band modulation compared with TD children, indicating altered oscillatory regulation during naturalistic ToM processing. Importantly, this group difference was not specific to the rTPJ, as the Condition × ROI × Group interaction was not significant. Moreover, exploratory analyses suggested that reduced rTPJ alpha-band modulation was associated with greater autism-related symptom severity, although this association did not survive FDR correction. Together, these findings support the utility of naturalistic EEG paradigms for studying ToM-related processing in children and identify alpha-band modulation as a preliminary neural correlate of individual differences in social-cognitive engagement.

Ecological Validity and Neural Specificity of the Naturalistic Paradigm

By combining a naturalistic movie-viewing paradigm with EEG, the present study aimed to move beyond static, instruction-driven laboratory tasks and characterize alpha-band activity associated with ToM-related processing under ecologically valid conditions. Naturalistic stimuli better approximate the dynamic, ambiguous, and continuous nature of real-world social interactions, which are often inadequately captured by traditional false-belief or instruction-based paradigms (Atherton et al., 2019, Richardson et al., 2018). The observed neural patterns therefore provide preliminary evidence for how scalp-level alpha-band modulation varies during naturalistic social-cognitive processing.

At the neural level, TD children showed increased alpha-band power during ToM-related scenes in the examined rTPJ and PFC ROIs, consistent with prior evidence implicating these regions as core nodes of the mentalizing network (Bowman et al., 2019, Ghuman et al., 2017, Symeonidou et al., 2020). However, the brain–behavior analyses showed a more cautious pattern: rTPJ alpha modulation showed an exploratory association with individual differences in caregiver-reported ToM abilities, as indexed by ToMI-2 scores, whereas PFC alpha modulation showed no such relationship. Notably, because this association was negative in direction, rTPJ alpha modulation should be interpreted cautiously as a correlational neural index of individual differences in ToM-related processing, rather than as a marker of better ToM ability. The negative direction of this association may reflect greater processing demands in children with lower caregiver-reported ToM abilities, compensatory engagement, differences in neural efficiency, or variability in caregiver-reported ToM measures.Moreover, because this association did not survive after FDR correction, it should be interpreted cautiously as an exploratory brain–behavior finding.

At the interpretive level, alpha-band activity has been proposed to be involved in inhibitory control over sensory-driven processing, enabling a shift toward internally oriented representations (Jensen and Mazaheri, 2010, Klimesch, 2012, Magosso et al., 2019, Sokoliuk et al., 2019). In naturalistic social contexts, ToM requires individuals to suppress competing perceptual input while constructing, maintaining, and updating internal models of others’ beliefs, intentions, and emotions. The broader alpha-band increase observed in TD children across the two a priori ROIs may be consistent with greater engagement of neural processes related to internally oriented social-cognitive processing that prioritizes internally generated social inferences over ongoing sensory information. This interpretation aligns with predictive processing accounts of social cognition, which emphasize continuous internal model updating under conditions of perceptual uncertainty (Zhou et al., 2023). In contrast, alpha modulation in the PFC may reflect more domain-general executive control processes, such as sustaining attention, maintaining narrative coherence, or regulating cognitive demands over time (Brooks et al., 2017, Funahashi, 2017). While these processes are necessary for successful engagement with complex stimuli, they are not specific to mental state attribution. The absence of a significant association between PFC alpha modulation and ToMI-2 scores is compatible with this interpretation, but does not establish functional specificity and suggests that PFC activity may play a supportive or supervisory role within the mentalizing network rather than directly indexing social inference computations.

Taken together, these findings suggest that the naturalistic paradigm engages functionally meaningful neural mechanisms relevant to real-world social cognition, while also exploratory ROI-level brain–behavior pattern within the ToM network. Importantly, the association between rTPJ alpha-band modulation and caregiver-reported ToM abilities should be interpreted cautiously, given its negative direction and the fact that it did not survive FDR correction. Rather than establishing a specific mechanism of internal attention during social inference, this finding suggests that rTPJ alpha-band modulation may be related to individual variability in naturalistic ToM-related processing.

### 4.1 Atypical neural activity in autistic children

Consistent with our second hypothesis, autistic children did not show clear context-dependent modulation of alpha-band activity during ToM-related scenes under naturalistic viewing conditions. Whereas TD children showed increased alpha-band modulation during ToM-related scenes in the examined rTPJ and PFC ROIs, autistic children did not show a comparable ToM-related change relative to baseline. This pattern suggests altered context-dependent alpha-band modulation in autism, rather than reduced absolute alpha-band modulation or reduced regional engagement per se. At the interpretive level, alpha-band oscillations have been linked to inhibitory gating and the regulation of internal versus external attention (Jensen and Mazaheri, 2010, Klimesch, 2012). In naturalistic social environments, mentalizing may involve the suppression of ongoing sensory input to allow internally generated representations—such as beliefs, intentions, and emotional states of others—to be constructed and updated. The absence of a comparable context-dependent alpha-band increase in autistic children may therefore be consistent with altered flexibility in shifting toward internally oriented social-cognitive processing when situational demands increase. This interpretation aligns with broader accounts of autism emphasizing atypical sensory weighting and altered top–down modulation during complex, real-world tasks (Mangnus et al., 2024, Quiñones-Camacho et al., 2021).

In the exploratory brain–behavior analyses, reduced rTPJ alpha modulation showed a negative association with autism-related symptom severity, as indexed by CARS scores, although this association did not survive after FDR correction. This pattern raises the possibility that altered alpha-band modulation may be related to heterogeneity in autism-related traits, but it does not establish a causal pathway linking neural dynamics to social–behavioral difficulties (Jurek et al., 2022, Pearcey et al., 2021). Notably, this association was observed for rTPJ alpha-band modulation but not for PFC alpha-band modulation. However, because the ROI-level correlations were not directly compared statistically, this pattern should not be interpreted as definitive evidence for regional specificity. The absence of a significant association between rTPJ alpha-band modulation and ToMI-2 scores in the ^autistic group^ further highlights the complexity of brain–behavior relationships in autism. Several factors may account for this null finding. First, autism is clinically heterogeneous, and children with similar caregiver-reported ToM scores may differ substantially in language ability, adaptive functioning, symptom profiles, intervention history, or compensatory strategies. Such heterogeneity may weaken a simple linear association between alpha-band modulation during passive movie viewing and everyday ToM abilities. Second, the ToMI-2 is a caregiver-report measure and may capture broad, context-dependent social-cognitive functioning in daily life rather than the specific neural processes engaged during brief naturalistic ToM-related scenes. Third, some autistic children may use compensatory behavioral strategies in everyday social situations, which could reduce the correspondence between caregiver-reported ToM abilities and task-related neural modulation. In addition, the rTPJ ROI was defined using scalp electrodes as an approximate region of interest rather than a precise anatomical source. Therefore, limited spatial specificity of the electrode-based ROI may have reduced sensitivity to individual differences in rTPJ-related cortical activity. In contrast, CARS reflects a broader constellation of social, communicative, and behavioral features, which may be more closely related to variability in naturalistic social-cognitive processing. This dissociation suggests that rTPJ alpha-band modulation should not be treated as a unitary or ToM-specific neural marker across groups; rather, it may reflect broader variability in social-cognitive engagement during naturalistic viewing.

Taken together, these findings suggest that autism is characterized by altered context-dependent alpha-band modulation during naturalistic ToM-related processing, rather than simply reduced absolute alpha-band modulation. Under naturalistic conditions, such altered modulation may be related to differences in internally oriented social-cognitive processing, but its developmental and behavioral significance requires further validation in larger, longitudinal, and independently replicated samples.

### 4.2 Implications

The findings of this study offer significant theoretical and practical implications. Methodologically, the findings suggest that naturalistic movie-viewing EEG may provide a feasible approach for characterizing alpha-band modulation associated with social–cognitive engagement in ecologically valid contexts. The scientific and clinical significance of this finding is twofold. For TD children, the findings may help characterize how alpha-band modulation during naturalistic ToM-related scenes relates to variability in caregiver-reported ToM abilities. However, because the rTPJ–ToMI-2 association was negative in direction and did not survive FDR correction, it should be regarded as exploratory rather than as evidence of a biomarker of social-cognitive functioning. In autistic children, altered context-dependent alpha-band modulation may similarly be related to heterogeneity in autism-related traits. The exploratory association between rTPJ alpha-band modulation and CARS scores suggests that this neural measure may be relevant to individual differences in autism-related symptom severity. Future work may examine whether such neural patterns can contribute to multivariate models of social-cognitive functioning, pending validation in larger, longitudinal, and independently replicated samples. If validated, such models may help characterize developmental heterogeneity in social-cognitive functioning, but the present data do not support automated classification or severity estimation based on rTPJ alpha modulation alone. Before these neural measures can be considered for clinical assessment or intervention-related outcomes, further work is needed to establish their reliability, specificity, predictive validity, and sensitivity to developmental or intervention-related change. Thus, the present study provides an initial step toward characterizing naturalistic EEG features associated with social-cognitive processing, while stronger claims regarding clinical identification, assessment, or intervention applications require further validation.

## 5 Limitations

The findings of this study should be interpreted in light of the following limitations. First, the sample composition has its limitations. The gender ratio in our autistic group was imbalanced, with a significant overrepresentation of males. It is important to note, however, that this ratio is consistent with the well-documented male prevalence in autism diagnoses, thus reflecting a real-world clinical demographic. Nevertheless, this imbalance, combined with recruitment from a single hospital outpatient clinic and a relatively homogenous community background, may still limit the broader generalizability of our findings. Future research should aim to recruit a more diverse and balanced sample through multiple channels, such as hospitals, schools and community organizations. Second, the clinical characterization of the autistic group was limited. Although autism diagnoses were established by experienced clinicians according to DSM-5 criteria and autism-related symptom severity was assessed using CARS scores, more detailed clinical information was not available for all participants. For example, language profiles, medication status, intervention history, adaptive functioning, and comorbid psychiatric or neurological conditions were available only when provided by clinical records or caregiver reports. Therefore, these factors could not be systematically included as covariates or moderators in the primary analyses. This limited autism characterization may have obscured clinically meaningful heterogeneity within the autistic group. Future studies should include more comprehensive and standardized clinical assessments to clarify how these factors may influence naturalistic ToM-related alpha-band modulation.

Third, the stimulus material itself had inherent limitations. While the naturalistic paradigm enhanced ecological validity, the specific stimuli used contained a finite number of explicit ToM events. This may have resulted in some aspects of ToM-related alpha-band modulation going unobserved.

Future work could benefit from constructing standardized stimulus libraries that more comprehensively simulate real-world social scenarios and provide sufficient event numbers for robust condition-level estimates. Fourth, the study did not cover the full spectrum of the autism population. We included only autistic children (IQ > 70), and the results may not be generalizable to autistic individuals with lower IQ scores, who often present with more severe cognitive and social deficits. Future studies should include children across different functional levels of autism to reveal potential neurological differences between subgroups. By addressing these limitations, subsequent research can advance the characterization of neurodevelopmental trajectories in autism and enhance the clinical applicability of research outcomes.

## 6 Conclusion

This study examined alpha-band activity associated with naturalistic ToM-related processing in young children using an ecologically valid movie-viewing EEG paradigm. The findings indicate that TD children showed context-sensitive alpha-band modulation during ToM-related scenes, whereas autistic children did not show clear modulation between ToM-related and baseline scenes. Exploratory brain–behavior analyses further suggested that rTPJ alpha-band modulation showed a negative association with caregiver-reported ToM abilities in TD children and was related to autism-related symptom severity in autistic children. However, these associations should be interpreted cautiously given the negative direction of the TD rTPJ-ToMI-2 association and the attenuation of the rTPJ associations after FDR correction. Overall, this work highlights the value of naturalistic EEG paradigms for characterizing alpha-band activity patterns associated with social-cognitive processing in early childhood, while stronger causal, mechanistic, diagnostic, or intervention-related interpretations require further longitudinal, experimental, and independently replicated evidence.

## List of abbreviations

TD: Typically Developing; ToM: Theory of Mind; rTPJ: Right Temporoparietal Junction; PFC: Prefrontal Cortex; EEG: Electroencephalography; CARS: Childhood Autism Rating Scale; ToMI-2: Theory of Mind Inventory–Second Edition

## Ethics approval

All participants provided written informed consent prior to participation in the study. This study was approved by the ethics committee of Shenzhen University Health Science Center (Approval Number: PN- 202200082) and Shenzhen Maternity and Child Healthcare Hospital (Approval Number：SFYLS[2022]080), and all procedures complied with the ethical standards of the 1964 Declaration of Helsinki regarding the treatment of human participants in research.

## Funding

The author(s) disclosed receipt of the following financial support for the research, authorship and/or publication of this article: This work was supported by the National Social Science Fund of China (21BYY111), Shenzhen Science and Technology Program (JCYJ20240813142626035 and JCYJ20240813115124032), Shenzhen Maternity and Child Healthcare Hospital (FYA2022018), the High-level Achievement Cultivation Project (24GSPCG06) and Medicine-Engineering Interdisciplinary Research Foundation of Shenzhen University (2023YG024).

## CRediT authorship contribution statement

**Hong Wang:** Writing – review & editing, Validation, Supervision, Resources, Project administration, Investigation, Funding acquisition, Conceptualization. **Tianqi Feng:** Investigation. **Zhen Wei:** Investigation. **Binbin Sun:** Writing – original draft, Visualization, Methodology, Formal analysis, Data curation, Conceptualization. **Xuefei Pan:** Writing – original draft, Visualization, Methodology, Formal analysis, Data curation, Conceptualization. **Shuo Zhao:** Writing – review & editing, Validation, Supervision, Resources, Project administration, Investigation, Funding acquisition, Conceptualization.

## Declaration of Competing Interest

The author(s) declared no potential conflicts of interest with respect to the research, authorship, and/or publication of this article.

## Data Availability

Data will be made available on request.

## Footnotes

Appendix A Supplementary data associated with this article can be found in the online version at doi:10.1016/j.dcn.2026.101810.
