# Supplementary material for: Altered alpha-band regulation during naturalistic theory of mind processing in autistic children
Source: Dev Cogn Neurosci. 2026 Sep 1;81:101810. doi: 10.1016/j.dcn.2026.101810 (PMC13572021; doi:10.1016/j.dcn.2026.101810)
Supplement: Supplementary file 1 [file mmc1.docx]

**Supplementary Material**

# **1. Supplementary Table**

# In this study, the film conditions included ToM conditions, Pain conditions and Baseline conditions. The specific time division is shown in Supplementary Table S1.

Supplementary Table S1. Three types of film conditions and time definition.

|  | Events | Time | Duration | Description |
| --- | --- | --- | --- | --- |
| ToM  condition | T1 | 1:04-1:10 | 6 | A crying baby becomes joyful upon receiving a helmet. |
|  | T2 | 1:18-1:26 | 8 | The scene shifts from white clouds to  the isolated-looking storm cloud Gus. |
|  | T3 | 1:40-1:44 | 4 | The stork Peck and Gus (friends) exchange greetings |
|  | T4 | 2:36-2:52 | 16 | Peck gazes longingly at a neighboring cloud crafting puppies,  prompting Gus to appear apprehensive, after which Peck notices his own envy and grows embarrassed. |
|  | T5 | 3:38-3:48 | 10 | Peck startles at the shark baby created by Gus  and observes another cloud producing adorable chicks. |
|  | T6 | 3:50-4:00 | 10 | Peck flies away from Gus,  landing on a nearby cloud that seems to mock Gus. |
|  | T7 | 4:32-4:46 | 14 | Peck dons protective gear to demonstrate loyalty to Gus,  requesting the cloud to craft a protective suit for himself.  Peek returns to Gus in protective gear,  showing through his actions that he did not leave. |
| Pian condition | P1 | 1:50-1:58 | 8 | Peck gets scared when he sees a baby crocodile and is bitten  when he makes a pass at the baby crocodile. |
|  | P2 | 2:20-2-26 | 6 | Peck steps back and Gazelle slams into Peck. |
|  | P3 | 3:00-3:16 | 16 | Peck delivers little hedgehogs that keep getting stabbed. |
|  | P4 | 3:20-3:28 | 8 | Peck got stung after delivering the baby hedgehog and lost lots of his feathers, then Gus helped Peck sort out his featherset. |
|  | P5 | 4:48-4:52 | 4 | Peck gets zapped by a baby electric eel. |
| Baseline condition | B1 | 0:00-1:04 | 64 | Introduction of white clouds, blue sky and other scenes. |
|  | B2 | 1:10-1:18 | 8 | The camera pans from a white cloud preparing a gift scene  to a dark cloud Gus. |
|  | B3 | 1:26-1:40 | 14 | Gus creates baby alligators. |
|  | B4 | 1:44-1:50 | 6 | Gus brought out a baby alligator to meet Peck. |
|  | B5 | 1:58-2:20 | 22 | Gus continued to work on the gazelle after Peck left. |
|  | B6 | 2:26-2:36 | 10 | Peck meets with a baby antelope. |
|  | B7 | 2:52-3:00 | 8 | Peck returns to Gus after dropping off the baby antelope. |
|  | B8 | 3:16-3:20 | 4 | Peck returns to Gus after dropping off the little hedgehog. |
|  | B9 | 3:28-3:38 | 10 | Gus meets Peck with a new gift. |
|  | B10 | 3:48-3:50 | 2 | White clouds and other scenes. |
|  | B11 | 4:00-4:32 | 32 | Scenes from the development of the story. |
|  | B12 | 4:46-4:48 | 2 | Gus brought out a baby eel to meet Peck. |
|  | B13 | 4:52-5:00 | 8 | Gus, Peck and the baby eels together. |

Supplementary Table S2. EEG preprocessing and data-quality indices by group.

| Measure | Autistic | TD | Group comparison  (t-test) |
| --- | --- | --- | --- |
| Number of excluded participants | 5 | 9 | - |
| Interpolated channels, mean ± SD | 5.78 ± 1.08 | 2.65 ± 0.95 | -13.171*** |
| Rejected ICA components, mean ± SD | 1.51 ± 0.51 | 0.71 ± 0.76 | -5.29*** |
| Retained ToM epochs, mean ± SD | 8.59 ± 3.46 | 7.38 ± 3.98 | -1.4 |
| Retained Baseline epochs, mean ± SD | 60.76 ± 10.37 | 27.15 ± 11.95 | -13.037*** |
| Retained total clean epochs, mean ± SD | 188.66 ± 13.83 | 223.47 ± 15.94 | 10.127*** |

*p* *** < 0.001.
